# Significance of intramedullary T2^*^ signal voids in the magnetic resonance imaging of paraplegic deep pain-negative dogs following intervertebral disc extrusion at short-term follow-up

**DOI:** 10.3389/fvets.2023.1248024

**Published:** 2023-09-13

**Authors:** Robert Clark, Amy Ferreira, Sebastien Behr

**Affiliations:** ^1^Neurology and Neurosurgery, Willows Veterinary Centre and Referral Service, Part of Linnaeus Veterinary Limited, Solihull, United Kingdom; ^2^Diagnostic Imaging, Willows Veterinary Centre and Referral Service, Solihull, United Kingdom

**Keywords:** myelomalacia, nociception, gradient echo, intervertebral disc extrusion, magnetic resonance imaging

## Abstract

**Introduction:**

Dogs presenting as paraplegic without nociception due to a thoracolumbar intervertebral disc extrusion provide a difficult decision to both the clinician and the owner. The prognosis when performing surgical decompression remains guarded. Aside from significant extradural compression, these dogs often have a significant secondary spinal cord injury, which has shown to be an important factor in determining both the likelihood of developing progressive myelomalacia and the return to ambulation.

**Materials and methods:**

This is a retrospective, observational, single centre study including 82 dogs presenting as paraplegic with absent nociception diagnosed with an intervertebral disc extrusion. Patients underwent MRI of the thoracolumbar spine, including a gradient echo sequence which was evaluated for the presence of intramedullary signal void artefacts. Decompressive surgery was performed, and patients were evaluated for the presence of nociception at short term follow up (at least four weeks post-surgery).

**Results:**

Overall, 59.8% of patients regained nociception within the study period. This number was significantly reduced to 33.3% when multiple gradient echo signal voids were present (compared to 67.3% of dogs without signal voids). There was no significant difference in the rate of developing progressive myelomalacia between groups.

**Conclusions:**

This paper adds to the existing literature and suggests that the gradient echo sequence may be of use when assessing acute spinal cord injury in the context of intervertebral disc extrusion and how it relates to prognosis.

## Introduction

Paraplegic deep pain-negative dogs and the possibility of progressive myelomalacia (PMM) provide a unique decision-making challenge to the veterinary surgeon and the client. The prognosis in dogs presenting as paraplegic with absent nociception remains guarded, with recovery rates reported to range from 30 to 75%, with an overall recovery rate of 61% (1–7). Previously, no correlation between the degree of compression and the initial neurological grade or eventual outcome had been found (8–10). This suggests that the degree of compression observed in dogs following an acute intervertebral disc extrusion (IVDE) may not be the most significant factor affecting the outcome. Research should therefore focus on intramedullary MRI changes in pre-surgery patients to better understand the probability of a positive or negative outcome.

PMM was first described clinically and histopathologically as the “ascending syndrome” in 1972. The authors identified extensive necrosis and ischaemic changes associated with marked extra and intramedullary haemorrhage of the spinal cords of eight dogs (11). PMM is characterised by progressive haemorrhagic necrosis of the spinal cord that ascends/descends over several spinal cord segments (12). More recently, dogs developing PMM have been demonstrated to exhibit an increase in biomarkers associated with oxidative stress (8-isoprostane F2α and acrolein), as well as decreased endogenous antioxidation of glutathione, thus suggesting that PMM may be the end point of cascading secondary injury that is unable to be regulated by the body following an acute spinal cord injury such as an IVDE (12). However, these tests are not available on the patient's side and thus cannot assist the clinician in decision-making prior to surgery.

Clinically, this condition is of great importance as PMM invariably proves fatal and can develop even after surgical decompression, with the majority of dogs being euthanised within 3 days of presentation (13).

PMM has been demonstrated to develop in dogs graded 3 (non-ambulatory paraparesis), 4 (paraplegic with intact nociception), and 5 (paraplegic with absent nociception) following an IVDE with a prevalence of 0.6, 2.7, and 14.5%, respectively (14). The prevalence of paraplegic deep pain-negative dogs developing PMM is generally accepted to be ~9–17.5% (4, 5, 15–17), although rates as high as 33% are reported in the French bulldog (2). Clinical factors associated with an increased risk of developing PMM in dogs include being <6 years of age, L5–L6 disc herniations, and rapidly progressive onset of clinical signs (2).

Despite the importance of identifying this condition prior to surgical decompression, few studies exist that assess the possible risk factors that may be identified on preoperative MRI. At present, the length of the T2-weighted hyperintensity within the spinal cord or the length of spinal cord swelling with the use of a HASTE sequence have been identified as risk factors for the development of PMM on preoperative MRI of the spinal cord. Initially, a T2-weighted hyperintensity longer than six times the length of the body of L2 was believed to be a characteristic of PMM (18). However, a more recent study concluded that a T2-weighted hyperintensity of more than 4.57 times the length of L2 was found in 84.6% of dogs with myelomalacia, and dogs with a T2-weighted hyperintensity over this length were 17.2 times more likely to develop myelomalacia (14). Dogs with a loss of CSF signal ratio of >7.4 times the length of L2 on HASTE sequences were shown to be more likely to develop PMM (19). Currently, to the authors' knowledge, one case report links the appearance of gradient echo signal void to histopathologically confirmed PMM post-mortem (20), and the presence of GRE signal void on preoperative MRI has not been evaluated in the context of short-term outcomes following surgery.

The pathology of naturally occurring acute spinal cord injury and PMM has previously been assessed, with varying degrees of intramedullary haemorrhage being identified (21). Gradient echo MRI creates a contrast between tissues based on T2^*^relaxation, which refers to the decay of transverse magnetisation caused by a combination of T2 decay (spin-spin relaxation) and the rate of net magnetization vector dephasing caused by external and local magnetic field inhomogeneities. Therefore, gradient echo or T2^*^-weighted imaging is able to highlight local inhomogeneities that can go undetected on spin echo sequences. T2^*^-weighted imaging is particularly sensitive to haemorrhage because certain haemoglobin breakdown products are paramagnetic and create local magnetic field inhomogeneities (22). Given that the main clinical application of the T2^*^-weighted GRE sequence is the identification of haemorrhage (23), it would stand to reason that this sequence would be useful when assessing haemorrhagic intramedullary spinal cord injury. The usefulness of GRE sequences has been evaluated retrospectively in canine and feline spinal cord diseases (24). This study did not evaluate intramedullary change, instead stating that the T2^*^-weighted GRE sequence may be useful in differentiating extradural haemorrhage from spinal cord parenchyma.

The presence of a GRE signal void within the spinal cord parenchyma has been shown to have a negative correlation with a successful outcome when managing an acute non-compressive intervertebral disc extrusion. However, this correlation was only significant when considering all dogs included in the study and became insignificant when grouping dogs by neurological grade (25). This highlights the possible importance of the GRE sequence as a measure of secondary spinal cord injury severity and its potential usefulness in assessing the risk of PMM.

Hence, this study aimed to evaluate the presence or absence of either single or multiple GRE signal voids on the preoperative MRI of patients presenting as paraplegic with absent nociception and their relationship to short-term outcomes. The hypothesis is that patients with single or multiple intramedullary GRE signal voids would be more likely to develop PMM and less likely to recover nociception.

## Materials and methods

### Study population

This was a retrospective, observational, single-centre study. Subjects were selected from dogs evaluated at Willows Veterinary Referral Hospital, Solihull between 2009 and 2021. The inclusion criteria were dogs referred for acute-onset paraplegia with absent nociception, as confirmed on neurological examination by an ECVN-certified or eligible veterinary neurologist or neurology resident under the supervision of an ECVN-certified or eligible veterinary neurologist. The sample size was determined by the number of patients evaluated during the study period. Patients must have undergone an MRI of the thoracolumbar spine, including at least T2-weighted sagittal, T2-weighted transverse, and T2^*^/GRE transverse sequences. Patients must have been diagnosed with intervertebral disc extrusions managed surgically via a hemilaminectomy or mini-hemilaminectomy. A follow-up period of at least 4 weeks with re-examination was required to confirm the presence or absence of PMM or nociception. The exclusion criteria were an incomplete MRI protocol (absence of the aforementioned sequences), the presence of MRI artefacts or a low-quality study, or insufficient follow-up information. Subject inclusion or exclusion decisions were made by an ECVN-certified veterinary neurologist (S.B.) and a resident in veterinary neurology (R.C.).

### Patient demographics and clinical assessment at presentation and re-examination

Individual medical records were reviewed, and the following data were recorded: age, breed, sex, presenting complaints and neurological examination findings, neuroanatomical localisation, and outcome following surgical intervention with a follow-up period of at least 4 weeks. The selected patients were divided into subgroups for comparison: patients recovering nociception vs. those not recovering nociception and patients developing suspected PMM vs. those not developing suspected PMM. A diagnosis of suspected PMM was made based on the following previously described clinical findings: loss of pelvic limb reflexes (patellar and withdrawal) and loss of perineal reflex when distant from the site of extrusion, poor abdominal wall tone, and cranial advancement of the cutaneous trunci reflex more than four sites cranial to the site of extrusion. Evidence of progressive-onset thoracic limb weakness, Horner's syndrome, or respiratory compromise was also considered consistent with PMM (13, 14, 16, 18, 19, 26). Loss of pelvic limb reflexes was differentiated from spinal shock when there was a loss of both the patella and withdrawal reflexes in extrusions cranial to L4–S1 spinal cord segments at the time of the initial examination.

### Image acquisition

Patients were anaesthetised as per protocols provided by the attending anaesthetist. All dogs were scanned using a standardised MRI protocol. Dogs were placed in dorsal recumbency for an MRI of the thoracolumbar spine using a 1.5-T system (Magentom Sola, Siemens Healthcare Limited, Erlangen, Federal Republic of Germany, or Signa HD, GE Healthcare, United States). Images were acquired in three standard planes, always including T2-weighted sagittal and transverse sequences and T2^*^-weighted GRE transverse sequences. In each case, a multichannel high-resolution spinal coil was used. When signal quality was considered poor, a multichannel high-resolution surface coil was used to improve image quality. Slice thickness ranged from 2.0 mm to 2.5 mm for T2-weighted sequences and from 2.0 mm to 3.5 mm for GRE sequences. The decision on which slice thickness to use was made by the attending radiographer at the time of imaging based on patient size (thicker slices for larger patients) and on the length of the spinal cord to be imaged (thicker slices for longer sections of the spinal cord).

### Image review

**Figure 1:**
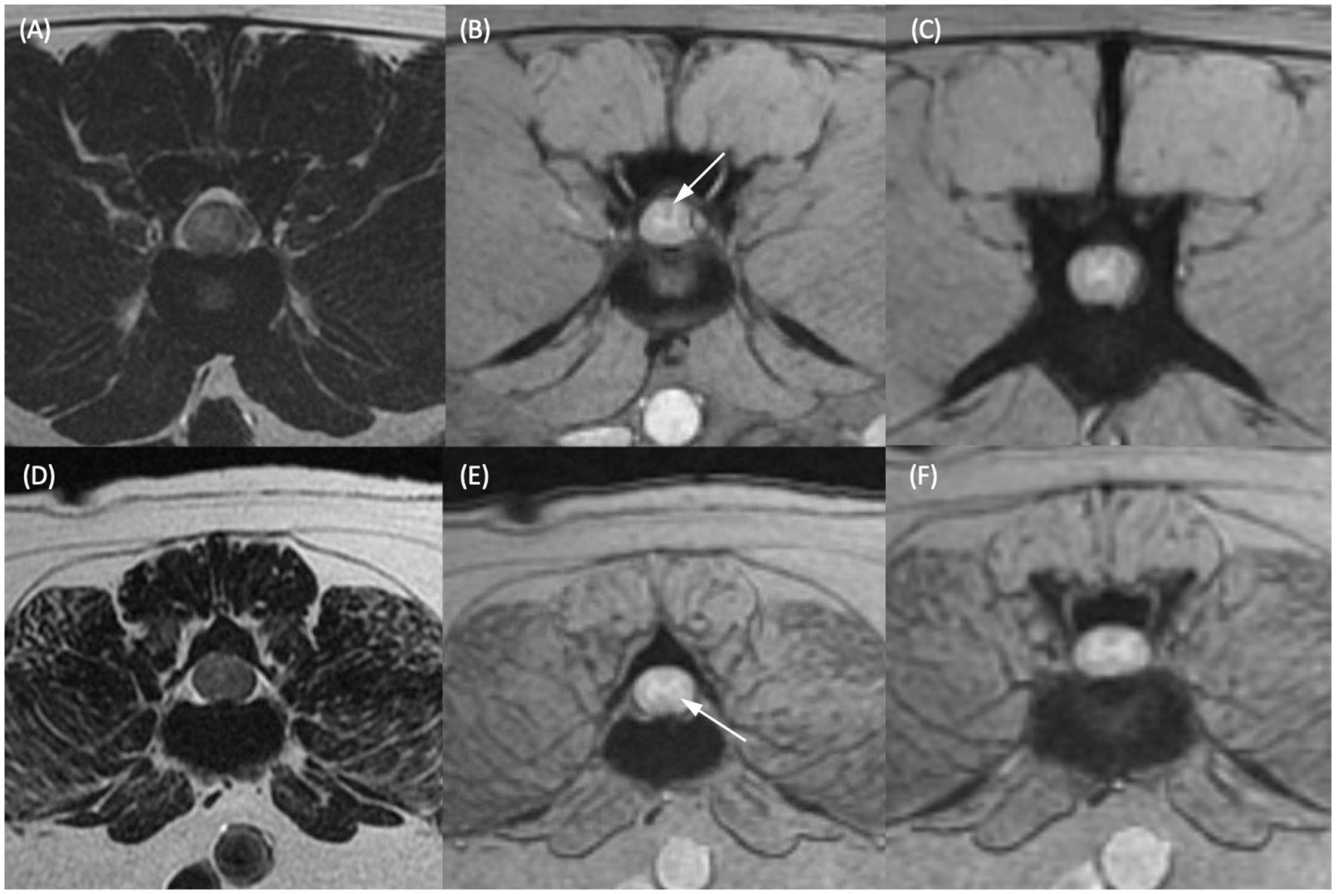
T2-weighted transverse **(A, D)** and T2*-weighted gradient echo (GRE) transverse **(B, C, E, F)** MR images of the vertebral column of two different dogs. **(A, B)** Dog 1 at the level of the L2–L3 intervertebral disc and **(C)** at the level of the cranial vertebral body of L4. **(D, E)** Dog 2 at the level of the caudal vertebral body of L3 and **(F)** at the level of the L3–L4 intervertebral disc. Within the dorsal funiculus **(B)** and left lateral funiculus **(E)** there are single, small, subtle, T2*-weighted GRE signal voids (white arrows). **(C, F)** Are given as regions of spinal cord considered not to have T2*-weighted GRE signal voids present in the same patients for comparison.

**Figure 2:**
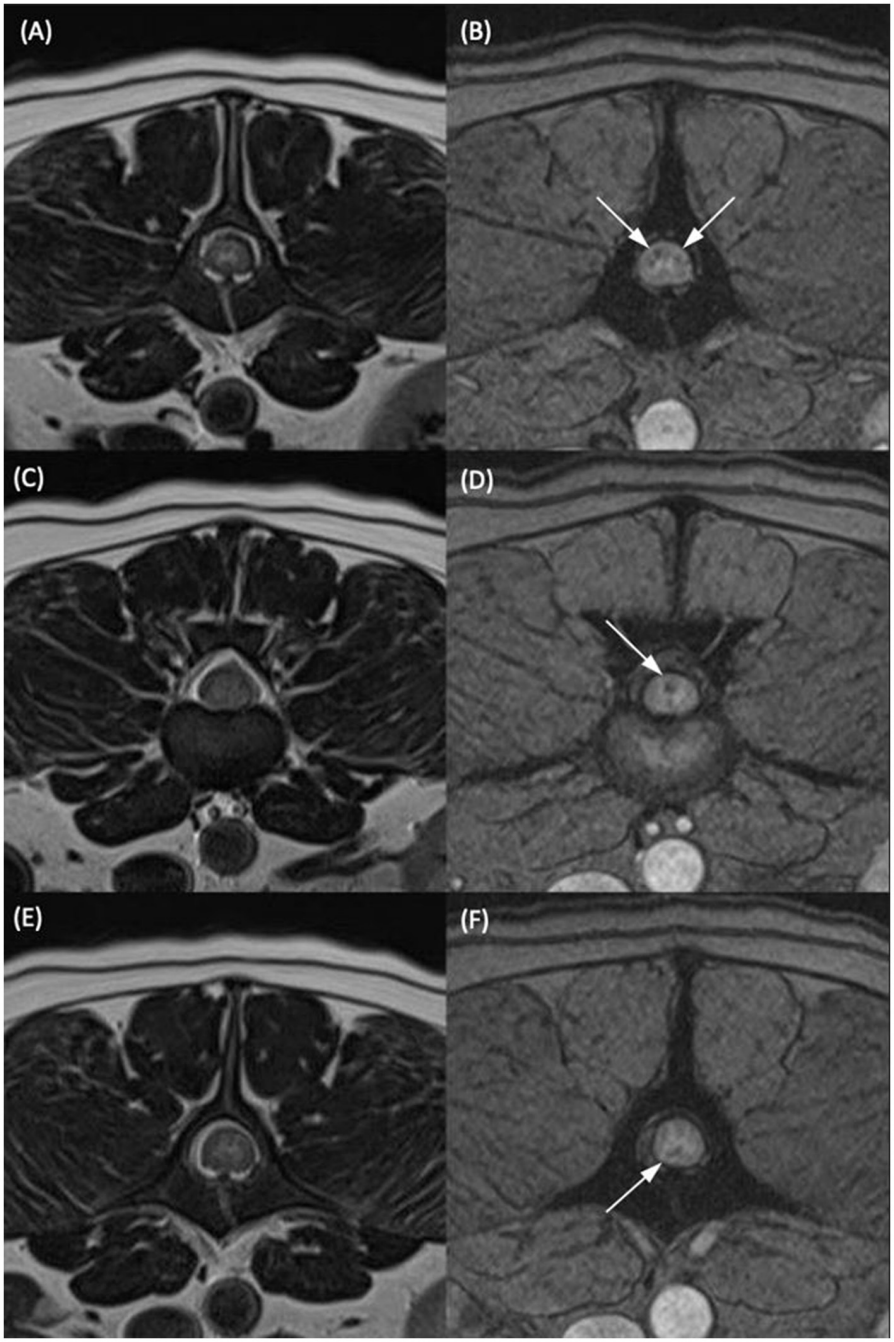
T2-weighted transverse **(A, C, E)** and T2*-weighted gradient echo (GRE) transverse **(B, D, F)** MR images of the vertebral column of the same dog classified as having multiple T2*-GRE signal voids. **(A, B)** At the level of the mid vertebral body of L2. **(C, D)** At the L2–L3 intervertebral disc space. **(E, F)** At the level of the cranial portion of the L3 vertebral body. Within the dorsal and both lateral funiculi **(B)**, the dorsal funiculus **(D)** and the ventral funiculus **(F)** were areas of T2*/GRE signal void. The signal voids were considered multiple as they were present in more than one region on one transverse image and present at several different levels in the same patient.

Images were reviewed by a European College of Veterinary Diagnostic Imaging (ECVDI) board-certified veterinary radiologist (A.F.) on DICOM viewing software (OsiriX, Pixmeo, Switzerland). The reviewer was aware that patients presented for paraplegia with absent nociception but was blinded to the outcome data. Hyperintensity of the spinal cord on T2-weighted sequences was defined as higher than that of the standard signal intensity of the spinal cord parenchyma at a distant and unaffected site. When a linear T2-weighted hyperintensity was visible on the mid-sagittal image, its longitudinal length was measured and expressed as a ratio when compared to the length of the L2 vertebral body (T2HI: L2 ratio). T2^*^/GRE signal void was defined as a region of susceptibility artefact that was hypointense to the standard signal intensity of the spinal cord parenchyma at an adjacent portion of the spinal cord. The GRE signal voids were denoted as being either single or multiple. Single GRE signal voids were defined as occupying a single area on a single transverse slice. Please see Figure 1 for two representative case examples of patients with a single GRE signal void. Multiple GRE signal voids were determined as two or more separate areas of signal void on one transverse image and/or GRE signal voids that were present on more than one transverse image (i.e., present at more than one level or large enough to occupy multiple slices). Figure 2 represents a single patient that was considered to have GRE signal voids occupying both different areas of the spinal cord on a single transverse slice but also having GRE signal voids present on multiple transverse slices.

### Statistical analysis

Statistical analyses were performed by one author (R.C.) using commercially available statistical analysis software (SPSS, IBM Corp., Released 2020. IBM SPSS Statistics for Macintosh, Version 27.0. Armonk, NY: IBM Corp). Individual datasets were compared using the chi-squared test or Fisher's exact test with statistical significance set at a *P-*value of <0.05. Binary regression was used when assessing the T2HI: L2 ratio's significance in relation to the recovery of nociception or the development of PMM. For all data, averages were expressed as a median with an interquartile range (IQR). Receiver operating characteristic (ROC) curves were performed to assess the correlation and most optimal sensitivity and specificity of T2HI: L2 when compared to the outcome. The data were then categorised based on this calculation to allow for categorical comparison and odds ratio calculations.

## Results

### Patient demographics

A total of 116 paraplegic dogs without nociception that had an MRI of the thoracolumbar spine presented within the study period. Of these, 31 were excluded due to immediate euthanasia following MRI (and hence insufficient follow-up information) and 3 due to a lack of GRE sequence available for analysis, leaving a total study population of 82 dogs. Breeds included Miniature Dachshunds [33], French Bulldogs (10), small cross breeds (9), Cocker Spaniels (6), Yorkshire Terriers (3), two each of Standard Dachshunds, Chihuahuas, Staffordshire Bull Terriers, Shih Tzus, Jack Russell Terriers, and one each of medium cross breeds, Toy Poodles, German Shepherds, Nova Scotia Duck Tolling Retrievers, Border Collies, Basset Blue de Gascoignes, Cockapoos, Chinese Crested dogs, Basset Hounds, Border Terriers, and Papillons. The median age of presentation was 4.8 years (IQR = 2.3 years). A total of 52 were male dogs (28 neutered) and 30 were female dogs (24 neutered).

### Prevalence of GRE signal voids

Within the study population, 21 dogs (25.6%) displayed multiple GRE signal voids, 12 dogs (14.6%) displayed single GRE signal voids, and 49 dogs (59.8%) did not display GRE signal voids.

### Recovery of nociception

A total of 59.8% (49 out of 82) of patients recovered nociception within the study period. Patients exhibiting multiple intramedullary GRE signal voids were significantly less likely to regain nociception within the study period (*P* = 0.004). A total of 33.3% of patients with multiple signal voids recovered nociception vs. 67.3% of those without signal voids. Patients with a single signal void recovered nociception in 75% of cases, though this did not reach statistical significance (*P* = 0.608) when compared to patients without GRE signal voids.

### Development of PMM

Moreover, 13.4% (11/82) of the total study population developed suspected PMM postoperatively. The presence of GRE signal voids (singular or multiple) was not significantly associated with the development of suspected PMM (P = 0.099 for multiple and *P* = 0.143 for single).

### Assessment of the T2HI:L2 ratio

The ROC analysis produced a cutoff value of 5.2 to produce a sensitivity of 81.8% and a specificity of 70% for the development of PMM, with the area under the curve measured at 0.712, deeming the predictive value acceptable. When this cutoff was used, dogs with a T2W length ratio of >5.2 were 2.35 times more likely (95% CI 0.736–3.667) to develop PMM.

## Discussion

Providing accurate prognostic information when evaluating paraplegic deep pain-negative dogs due to an IVDE represents a clinical challenge. The outcome following surgery for this cohort of dogs is consistent with previous results published in the literature based on the severity of the clinical examination (1, 3–7, 15). Reliable information pertaining to prognosis as determined by changes on an MRI is limited. The relative sensitivity and specificity of T2wHI:L2 and SSTSE sequence evaluation are considered low (18, 19), and new imaging features that inform on prognosis would prove invaluable in the selection of surgical cases and setting client expectations.

In human medicine, the presence of intramedullary spinal cord haemorrhage has been consistently associated with severe spinal cord injury and a worse prognosis (27). This has been investigated in naturally occurring spinal cord injury in dogs in the context of an intramedullary T2-weighted hypointensity representing haemorrhage and demonstrating a worse prognosis in dogs with intramedullary haemorrhage (28). The usefulness of GRE sequences in the imaging of canine and feline spines has been previously investigated. However, there were no cases in which intramedullary signal voids in the acute stages following an intervertebral disc extrusion were demonstrated (24). Only one published case report assesses the appearance of PMM when performing GRE sequences (20). The MRI appearance (including eight dogs with T2^*^-weighted GRE sequences) of the spinal cord in dogs with incomplete recovery after severe spinal cord injury has been assessed. There was no evidence of intramedullary haemorrhage (as identified by signal voids) present, though all the dogs included had MRIs at least 3 months following the initial injury (29). To the authors' knowledge, this is the first publication documenting the use of the GRE sequence in the context of acute spinal cord injury due to IVDE in dogs prior to surgery.

In the context of severe, acute spinal cord injury, there is little evidence comparing the appearance of the spinal cord on the GRE sequence to histopathological changes. In one case of a dog suffering from PMM, MRI demonstrated a severe and diffuse intramedullary GRE signal void artefact. When evaluated histopathologically, there was extensive liquefactive necrosis of the white matter with multifocal areas of haemorrhage at the junction between malacic and degenerated white matter (20). Although this represents an extreme example, given the noted histopathological appearance of areas displaying the GRE signal voids described, it would stand to reason that a similarly damaging process would occur in our dog populations, worsening their prognosis.

Multiple GRE signal voids were associated with a worsening prognosis, with only 33.3% of patients with multiple signal voids regaining nociception in comparison with 67.3% of patients without GRE signal voids in this study. Although this figure may not be used individually to predict the outcome, in the context of other previous imaging findings demonstrated to be associated with poor prognosis (6, 13), it may assist the clinician in setting client expectations. The presence of GRE signal voids failed to reach significance in the context of predicting PMM, though this may be due to a type II statistical error arising from low case numbers (n = 11). As per our previously outlined exclusion criteria, a number of dogs (n = 31) were excluded due to immediate euthanasia following an MRI. Had these patients not been euthanised, their inclusion may have altered the results.

One potential weakness in the way in which GRE signal voids were assessed in this study was that the classification did not allow for assessing the true extent of the voids reported (either in cross-sectional area or longitudinal length). When using GRE to identify small lesions (microbleeds) in human medicine, there was poor to moderate interobserver agreement (30). By differentiating single vs. multiple signal voids, an attempt was made to reduce the subjectivity present in the interpretation of the MRI studies and to identify lesions more objectively. Due to the potential implications of a patient being identified as having intramedullary haemorrhage (i.e., euthanasia following MRI in the clinical setting), it felt appropriate to differentiate the patients with more objective change and presumably more severe spinal cord injury.

The retrospective nature of the study was considered to have several limitations, including the accurate acquisition of data and patient enrolment. Paramount to this was the euthanasia of 31 patients under general anaesthesia following MRI. Within this population may have been patients considered to have the most severe MRI changes (as measured by T2-weighted hyperintensity, swelling of the spinal cord, and intramedullary GRE signal voids), with the inclusion of these patients perhaps altering our results. Unfortunately, the outcome of this population could not be assessed and was hence excluded from the current study.

When considering previously assessed MRI characteristics within our study population, it was found that a longer T2-weighted hyperintensity to L2 length ratio was significantly associated with developing PMM, as has been previously described. This prior publication considered dogs of all neurological grades and was performed on low field MRI images (14).

When assessing the outcome of this study, it was decided to use the recovery of nociception in the short term, although, ultimately, this does not mean the return of ambulation for all patients. In a previous study, 15% of patients regaining nociception failed to regain ambulation within a 12-week period. However, all were reported to have good voluntary movements (17). The selected outcome did allow for a greater number of patients to be included and for more objective data collection. The postoperative assessment of nociception and clinical presentation of PMM was always performed by an ECVN-certified or board-eligible neurologist or a neurology resident under the supervision of an ECVN-certified or board-eligible neurologist. A further prospective study in this area may generate adequate data to analyse the return to ambulation and gait analysis over long-term follow-up.

In conclusion, paraplegic dogs with absent nociception due to an IVDE were significantly less likely to recover nociception by short-term follow-up when multiple intramedullary GRE signal voids were identified. Based on these results, the authors would recommend the addition of this sequence in paraplegic deep pain-negative dogs due to IVDE to the standard T2-weighted sagittal, dorsal, and transverse sequences typically acquired to allow for a more accurate assessment of severe secondary spinal cord injury. Although no significant link was drawn between the presence of intramedullary GRE signal voids and the development of PMM, further prospective studies that include a more objective assessment of the nature of the intramedullary GRE signal voids with a larger number of dogs developing PMM may provide further insights.

## Data availability statement

The raw data supporting the conclusions of this article will be made available by the authors, without undue reservation.

## Ethics statement

Ethical approval was not required for the studies involving animals in accordance with the local legislation and institutional requirements because the study was retrospective and owners were not contacted. Written informed consent was obtained from the owners for the participation of their animals in this study.

## Author contributions

RC: study design, data acquisition, statistics calculation, and manuscript preparation. AF: study design, image review, data acquisition, and review of the final manuscript. SB: study design, data acquisition, and review of the manuscript. All authors contributed to the article and approved the submitted version.
